# Meiotic Silencing in Pigs: A Case Study in a Translocated Azoospermic Boar

**DOI:** 10.3390/genes12081137

**Published:** 2021-07-27

**Authors:** Nicolas Mary, Anne Calgaro, Harmonie Barasc, Nathalie Bonnet, Stéphane Ferchaud, Isabelle Raymond-Letron, Alain Ducos, Alain Pinton

**Affiliations:** 1 GenPhySE, Université de Toulouse, INRAE, ENVT, 31326 Castanet Tolosan, France; nicolas.mary@envt.fr (N.M.); anne.calgaro@envt.fr (A.C.); harmonie.barasc@envt.fr (H.B.); nathalie.bonnet@envt.fr (N.B.); alain.ducos@envt.fr (A.D.); 2 GenESI, UE 1372 Génétique, Expérimentations et Systèmes Innovants, 86480 Rouillé, France; stephane.ferchaud@inrae.fr; 3 STROMA Lab, Université de Toulouse, Inserm U1031, CNRS ERL 5311, EFS, ENVT, UPS, 31000 Toulouse, France; isabelle.raymondletron@envt.fr

**Keywords:** infertility, meiosis, reciprocal translocation, MSUC, MSCI (meiotic sex chromosome inactivation) disturbance

## Abstract

Carriers of balanced constitutional reciprocal translocations usually present a normal phenotype, but often show reproductive disorders. For the first time in pigs, we analyzed the meiotic process of an autosome–autosome translocation associated with azoospermia. Meiotic process analysis revealed the presence of unpaired autosomal segments with histone γH2AX accumulation sometimes associated with the XY body. Additionally, γH2AX signals were observed on apparently synapsed autosomes other than the SSC1 or SSC15, as previously observed in Ataxia with oculomotor apraxia type 2 patients or knock-out mice for the Senataxin gene. Gene expression showed a downregulation of genes selected on chromosomes 1 and 15, but no upregulation of SSCX genes. We hypothesized that the total meiotic arrest observed in this boar might be due to the silencing of crucial autosomal genes by the mechanism referred to as meiotic silencing of unsynapsed chromatin (MSUC).

## 1. Introduction

It is now well known that abnormalities in chromosome pairing during the first meiotic division (asynapsis) are associated with reduced fertility, particularly in male mammals [1,2]. Chromosomal anomalies can cause asynapsis, which activates checkpoints that arrest meiotic cells through apoptosis of pachytene spermatocytes and then reduce the production of gametes. Thus, the prevalence of reciprocal translocations is about 10 times higher in infertile men, some of whom have abnormal spermatogenesis [3]. In humans and mice, transcriptional silencing of asynapsed chromosomes or chromosome regions is one consequence of asynapsis [4,5,6]. As proposed by Turner [7], this meiotic silencing consists of a chromatin remodeling process in which genes located along unsynapsed chromosomes are transcriptionally silenced during meiotic prophase I. This meiotic silencing of unsynapsed chromatin (MSUC) [8] is characterized by the accumulation of BRCA1 and ATR recruited by SYCP3 and HORMAD1/2 on asynapsed regions, the spreading of ATR into the associated chromatin loops, and the phosphorylation of H2AX [4,5]. This phenomenon results from the signaling of asynapsis by some sensors (SYCP3, SMC1b, CDK2 HORMAD 1 and 2 and components of the BRCA1-A complex) and the genic inactivation by some effectors (e.g., MDC1, Senataxin AGO4, RAD18, HR6B and the variant histone H2AX) (Tuner, 2015). Senataxin, a DNA/RNA helicase involved in transcription regulation, RNA processing, and protection of the genome against oxidative damage, is essential for male meiosis, implicated in the process of meiotic sex chromosome inactivation (MSCI) as well as in the interface of transcription and meiotic recombination [9]. Indeed, meiosis in *Setx−/−* male mice presented spermatocytes arrested at the pachytene stage of meiotic prophase. The same phenotype (disruption of the spermatogenesis characterized by an arrest of meiosis at the spermatocyte stage) was observed in ataxia with oculomotor apraxia type 2 patients who carried a mutation of the *SETX* gene [10,11]. Moreover, in *Setx−/−* male mice, γH2AX foci were observed on apparently synapsed autosomes, indicating the persistence of unrepaired DSBs. Gene expression analysis also revealed a defective meiotic sex chromosome inactivation (MSCI) in the *Setx-* spermatocytes [9].

MSCI is a natural process of transcriptional silencing of the X and Y chromosomes that occurs during the meiotic phase of spermatogenesis. When the autosome synapsis is completed (zygotene-to-pachytene transition), the X and Y chromosomes are silenced and compartmentalized into a nuclear subdomain called the sex- or XY body [12,13]. The X and Y chromosomes (active during spermatogonial divisions and early meiotic stages) are then silenced from pachytene until the end of spermatogenesis [14]. Asynapsis due to chromosomal anomalies can be responsible for the association of the rearranged chromosomes with the XY bivalent. This association interferes with meiotic sex chromosome inactivation (MSCI) and disturbs transcriptional inactivation of the sex chromosomes [15]. It can also lead to the spreading of XY body inactivation towards the autosomal segments attached to the XY body, without reactivation of the latter.

The association of the XY bivalent and rearranged autosomes was observed in mice and humans [16,17,18,19,20,21,22,23,24,25,26,27,28,29], as well as in other mammals; see for instance [30,31,32]. The frequency of this association increases along the pachytene substages and reaches a maximum at late pachytene [28].

In pig species, the national systematic controls of young boars carried out in France made it possible to estimate the prevalence of balanced structural chromosomal rearrangements at a 0.5% level [33]. Among them, reciprocal translocations are the most frequent (87%) and, in the majority of the cases, are not associated with spermatogenesis impairment. Indeed, until now, only one case of autosome–autosome reciprocal translocation associated with impaired fertility has been reported [31]. We report, for the first time, a case of a reciprocal translocation in pigs involving autosomes which are responsible for the total arrest of spermatogenesis. The aim of this article was to document the meiotic silencing process in pigs by analyzing the early stages of meiosis in this infertile boar.

## 2. Materials and Methods

### 2.1. Animal

The reciprocal translocation t(1;15) was identified using classical cytogenetic analysis (GTG banding) during national systematic controls of young boars to be used in artificial insemination centers. The boar (Pietrain breed) was phenotypically normal but presented azoospermia: no spermatozoa detected among eight different ejaculates.

In accordance with the European Directive 2010/63/UE on the protection of animals, the procedure for testis collection was approved by the Ethics Committee of the Poitou Charentes region (France) (CE2012-2), under agreement number A-17-661. Testicular samples were collected by surgical castration.

### 2.2. Molecular Characterization of Breakpoints

The breakpoint position was determined by FISH using Bacterial Artificial Chromosome (BAC) clones obtained from the INRAE @BRIDGe Platform (http://abridge.inra.fr/index.php, 1 April 2020) [27]. The BACs SBAB-91F6 (located on SSC1) and CH242-4A8 (located on SSC15) were labeled with biotin using the BioPrime DNA Labeling System kit (LIFE TECHNOLOGIES, Carlsbad, CA, USA) and revealed by Alexa 594 conjugated to Streptavidin (MOLECULAR PROBES; Eugene, OR, USA). The BAC CH242-29P21 (SSC15) was labeled by digoxygenin and revealed using FITC conjugated mouse anti-digoxygenin antibodies (Sigma, St. Louis, MO, USA), as previously described [31].

### 2.3. Histopathological Analysis of the Testis Sample

Testis samples were fixed in 10% buffered formalin for 48 h before routine processing. Four-µm-thick paraffin sections were stained with hematoxylin and eosin.

Immunohistochemistry was performed with an active Caspase3 rabbit polyclonal antibody (ref. AF 835, R&D SYSTEMS, Minneapolis, MN, USA) as already described [34] and with an anti PGP9 mouse monoclonal antibody (ref. ab8189, ABCAM, Cambridge, UK).

Briefly, 4-micrometer paraffin-embedded sections from a testis were dewaxed in toluene and rehydrated first in alcohol bath and then in deionized water. Antigen retrieval was carried out in 10 mM citrate buffer, pH 6.0, for 30 min in a water bath at 95 °C. The cooled sections were then incubated in Dako peroxidase blocking solution (DAKO, Glostrup, Denmark) to quench any endogenous peroxidase activity. Nonspecific binding was blocked by incubation in normal goat serum at 1:10 dilution (DAKO, Glostrup, Denmark) for 20 min at room temperature (RT). The primary antibody was anti-active caspase-3 (dilution 1:300) (R&D SYSTEMS, Minneapolis, MN, USA) or anti-PGP9 antibody (dilution 1:1000) (ABCAM, Cambridge, UK). Sections were incubated with primary antibodies for 50 min at RT. Bound primary antibodies were detected with EnVision™ + Horse Radish Peroxidase (HRP) Systems (DAKO, Glostrup, Denmark) for 30 min at RT. Peroxidase activity was revealed by 3,3′-diaminobenzidine tetrahydro-chloride substrate (DAKO, Glostrup, Denmark). Finally, the sections were counterstained with Harris hematoxylin, dehydrated and coverslipped.

### 2.4. Analysis of Meiotic Pairing

#### 2.4.1. Immunocytology

Meiotic pairing analysis was carried out using two different immunocytology approaches.

Meiotic cells were prepared as previously described [31,35].

Briefly, meiotic proteins were immunolocalized using antibodies at 1:100 dilution in PBT (1X phosphate-buffered saline (PBS), 0.15% bovine serum albumin (BSA), and 0.1% Tween 20), as follows.

In a first experiment, the SCP3 and SCP1, the centromeres and the γH2AX proteins were detected using rabbit anti-SCP3 and rabbit anti-SCP1 (ABCAM, Cambridge, UK), human anti-centromere (ANTIBODIES INCORPORATED, Davis, CA, USA) and mouse anti-γH2AX (ABCAM, Cambridge, UK) antibodies. Secondary antibodies consisted of Dylight 594 conjugated goat anti-rabbit (KPL, Gaithersburg, MD, USA) and 1-amino-4-methylcoumarin-3-acetic acid (AMCA) conjugated donkey anti-human (JACKSON IMMUNORESEARCH LABORATORIES, Grove, PA, USA) and Alexa 488 conjugated goat anti-mouse (MOLECULAR PROBES, Eugene, OR, USA).

In a second experiment, a sequential detection of SCP1 and SCP3 was carried out. For this, the SCP1 and centromeres were first detected using the following primary antibodies: rabbit anti-SCP1 (ABCAM, Cambridge, UK) and Human anti-centromere (ANTIBODIES INCORPORATED, Davis, CA, USA). Secondary antibodies consisted of Dylight 488 conjugated goat anti-rabbit (KPL, Gaithersburg, MD, USA) and 1-amino-4-methylcoumarin-3-acetic acid (AMCA) conjugated donkey anti-human (JACKSON IMMUNORESEARCH LABORATORIES, Grove, PA, USA). SCP3 was subsequently detected using rabbit anti-SCP3 (ABCAM, Cambridge, UK) and then revealed with secondary antibody Alexa 594 conjugated donkey anti-rabbit (MOLECULAR PROBES, Eugene, OR, USA). Spermatocytes were captured using a Zeiss Imager Z2 microscope with the Cytovision imaging system (LEICA MICROSYSTEMES, Nanterre, France).

#### 2.4.2. Fluorescence In Situ Hybridization

After SC analysis, the same cells were subjected to fluorescence in situ hybridization with BAC clones: one was located in the telomeric region of the SSC1 p-arm (labeled with biotin), and one in the centromeric region of SSC15. These BAC clones were labeled using the BioPrime DNA Labeling System kit (LIFE TECHNOLOGIES, Carlsbad, CA, USA), and revealed by Alexa 594 conjugated to Streptavidin (MOLECULAR PROBES, Eugene, OR, USA) and fluorescein isothiocyanate (FITC) conjugated mouse anti-digoxygenin antibodies (SIGMA-ALDRICH, St. Louis, MO, USA). FISH signals from the same cells for which SCs had previously been analyzed were captured and evaluated using an Imager Z2 microscope with the Cytovision imaging system (LEICA MICROSYSTEMES, Nanterre, France).

### 2.5. Gene Expression Analysis

The impact of synapsis impairment was investigated by analyzing the expression of some SSC 1 and SCC15 genes located around the supposed breakpoint. These genes were selected due to their function in the meiosis or spermatogenesis process. Some SSCX genes were also analyzed to study the impact of the rearrangement on the MSCI process (Table S1). Total RNAs were extracted from testicular tissue of the t(1;15) carrier and three fertile control boars with normal semen parameters. Three independent extractions from three samples were performed for each animal using the Nucleospin RNA plus (Macherey-Nagel, Düren, Germany). The quality of RNA from all samples was established using the high RNA integrity numbers (RIN ≥ 8.8) on the 2100 Bioanalyzer (Agilent, Les Ulis, France), and concentrations were measured using a Clariostar (BMG Labtech, Champigny s/Marne, France). One μg of total RNA from each sample was reverse-transcribed using the qScript cDNA SuperMix (Quantabio, Beverly, MA, USA).

All primer sets were designed based on the genomic data of *Sus scrofa* (assembly Sscrofa11.1). A total of 20 gene primer pairs were designed, including three reference genes in two exon junctions to specifically amplify the transcripts. All primers were synthesized by Sigma Aldrich (Saint-Quentin Fallavier, France) and tested with four different concentrations (300/300, 300/900, 900/300 and 900/900 nM) to determine their optimal concentrations in the mix. Primer validation was carried out with the best amplification curve. Dissociation curves and Sanger sequencing of the qPCR products were used to confirm the good amplification of each gene. Negative controls in which no primers were added and a no template control were included to control reagent contamination. Primer sequences are listed in Table S1.

Experiments were carried out using a ViiA7 Real-Time PCR System (Applied Bio-systems, Foster City, CA, USA). The 384 well plates were prepared by an Agilent Bravo Automated Liquid Handling Platform (Agilent Technologies, Santa Clara, CA, USA). The samples were run in duplicate in a 5 µL reaction consisting of 1× of Power SYBR Green PCR Master Mix (Applied Biosystems, Foster City, CA, USA), 2.5 ng of cDNA, and 300 to 900 nM of primers (Table S1). Real-Time PCR was performed under the following thermal profile: a first one-hold stage at 95 °C for 10 min, followed by 40 cycles (95 °C for 15 s and 60 °C for 30 s), and a final extending step generated between 65 °C to 95 °C in 0.05 °C/s increments for melt curve analysis. The results were analyzed with Quant-Studio Real-time PCR software v1.1 (Applied Biosystems, Foster City, CA, USA). Housekeeping genes (GAPDH, β-Actin and RPL4) were analyzed with the Normfinder algorithm [36], and GAPDH was selected as the most stable normalizer against each target gene and used to calculate their relative expressions. Target-specific PCR efficiencies were calculated by averaging the individual amplification curve-based values determined by LinRegPCR software [37]. Target expression levels were determined by the efficiency-corrected ΔΔCt method and statistically compared to the control by a Student’s *t*-test.

## 3. Results

### 3.1. Cytogenetic and Molecular Characterization of Breakpoints

**Figure 1:**
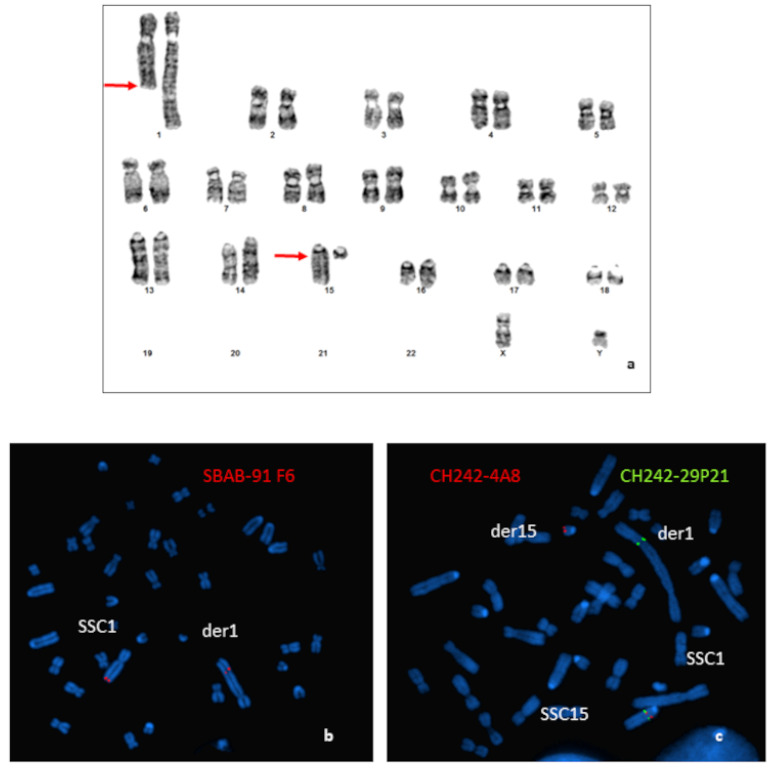
Characterization of the chromosomal rearrangement. (**a**) GTG-banded karyotype of the boar carrying a t(1;15)(q2.11;q1.2) translocation. Arrows indicate the locations of the breakpoints. (**b**) Localization of the breakpoints by FISH using BAC clones: (**b**) hybridization of the SBAB-91F6 (red); (**c**) hybridization of the CH242-4A8 (red); and CH242-29P21 (green) BAC clones.

Cytogenetic analysis from GTG-banded karyotypes of an azoospermic boar revealed a balanced reciprocal translocation between the q arm of one SSC1 and the q arm of one SSC15 (Figure 1a). The SSC1 breakpoint was very distal (q2.11 band), whereas the breakpoint on the acrocentric SSC15 was close to the centromere (q1.2 band). Characterization was completed by FISH.

Results obtained for the more distal BAC probe located on the SSC1 (SBAB-91F6: 274,154,166–274,274,406 bp on the Sscrofa11.1) revealed that the breakpoint was located between the end of the BAC and the end of the chromosome (Figure 1b).

Hybridization of different BAC clones located in the peri-centromeric region of the SSC15 revealed that the breakpoint was localized between the BACs CH242-4A8 (31,208,879–31,409,150 bp on the Sscrofa11.1) and CH242-29P21 (32,619,596–32,804,821 bp on the Sscrofa11.1) (Figure 1c).

### 3.2. Histological Analysis

**Figure 2:**
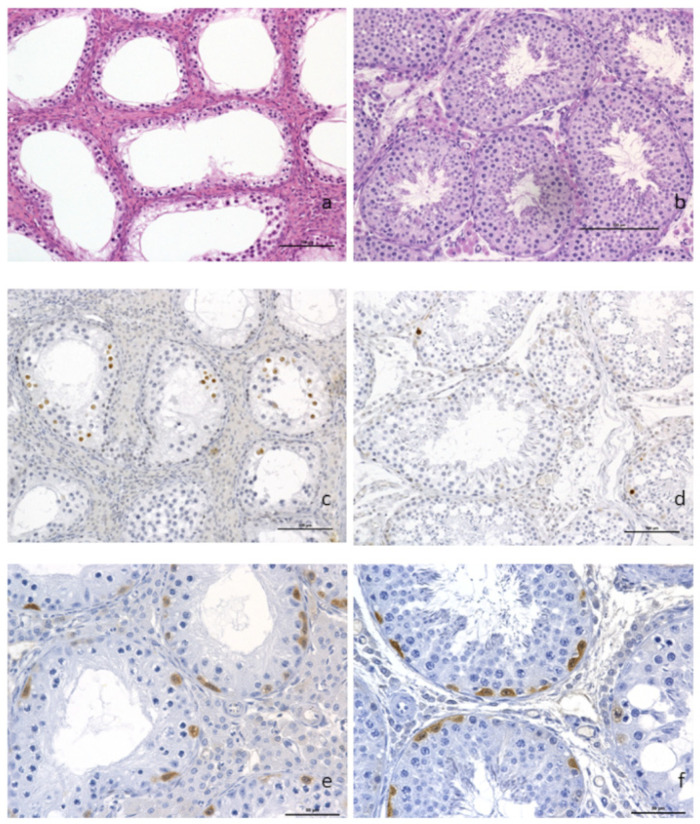
Histopathological analysis of testicular tissue. (**a**) Complete arrest of spermatogenesis with hyperplasia of the Leydig interstitial cells (×200) and presence of spermatogonia and primary spermatocytes but no evidence of further cell maturation. (**b**) control boar with normal spermatogenesis. Anti-caspase-3 immunohistochemistry on paraffin sections from t(1;15) (**c**) and control (**d**) testis samples (×200). A higher proportion of positive spermatocytes (dark brown staining of the nuclei) was observed in the testis of the translocated boar as compared to the control. Labeling of undifferentiated spermatogonia using PGP9.5 antibodies, translocated boar and (**e**) control (**f**) (×400). Scale bars are equal to 100 μm (**a**–**d**) and 50 μm (**e**,**f**).

Plasmatic levels of LH (Luteinizing Hormone), progesterone, testosterone and androstenedione were within normal ranges (1 ng/L, 2 nmol/L, 24 to 55 nmol/L, and 1.3 to 3.4 nmol/L, respectively). However, routine histopathological evaluation of the testis showed a diffuse and severe tubular atrophy, illustrated by a marked thinning and decrease in cell density of the tubular wall linked to a complete arrest of spermatogenesis. Only spermatogonia and primary spermatocytes were observed, but there was no further cell maturation of the seminiferous tubule lining. A diffuse moderate hyperplasia of the interstitial Leydig cells was also observed, as was the complete spermatocyte vacuity of the epididymis (Figure 2a).

Immunohistochemical identification of apoptotic cells with anti-caspase 3 antibody highlighted an increased number of spermatocytes with nuclear brown labeling in the testis of the translocated boar (Figure 2b), compared to the control boar testis (Figure 2c). The germinal epithelium basal layers of the tubules showed the presence of spermatogonia and primary spermatocytes in great number, as demonstrated by the anti-PGP9.5 brown cytoplasmic labeling of undifferentiated spermatogonia (Figure 2d). PGP9.5-positive spermatogonia were observed in the same proportion as on the normal control boar testis (Figure 2e).

### 3.3. Analysis of Meiotic Pairing

**Figure 3:**
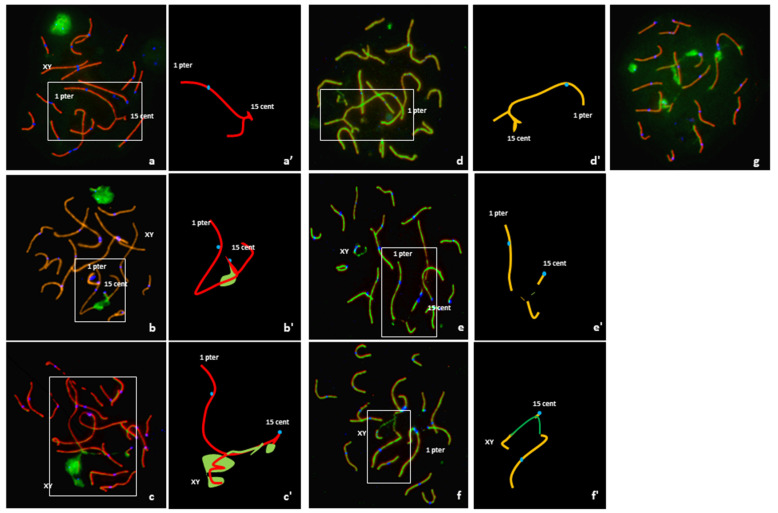
Analysis of meiotic pairing in pachytene spermatocytes using FISH and immunostaining of the synaptonenal complex proteins SCP1 (red) + SCP3 (red), γ H2AX (green) and the centromeres(blue) and graphical representation of quadrivalents. (**a**,**a’**) Complete synapsis, no γ H2AX signal on the quadrivalent, and no association with the XY body. (**b**,**b’**) Unsynapsed segment with γ H2AX signal on the quadrivalent but no association with the XY body. (**c**,**c’**) Association of the quadrivalent with the XY body; spreading of the γH2AX along the quadrivalent. (**d**,**d’**) Quadrivalent with fully synapsed, i.e., presence of the SC lateral and central elements all along the SSC1 and SSC15 axis (SCP1 and SCP3 revealed in green and red, respectively). (**e**,**e’**) Unsynapsed quadrivalent with no association with the XY body. (**f**,**f’**) Association between the quadrivalent and the XY body. (**g**) γH2AX signals on different apparently synapsed autosomes.

An analysis of 108 pachytene nuclei showed three different pairing patterns between normal and rearranged SSC1 and SSC15 (Table 1). In 12% of the cells, these chromosomes were perfectly paired in a quadrivalent configuration with no positive γH2AX signal on the corresponding autosomal regions (Figure 3a). Conversely, a positive γH2AX signal on the quadrivalent was observed in 88% of the cells (Figure 3b). In 34% of these spermatocytes, the γH2AX signals were observed on the quadrivalent, and in 54%, with an association with the XY body (Figure 3c).

It is important to note that of the 108 spermatocytes analyzed, 54% presented γH2AX signals on apparently synapsed autosomes other than the SSC1 or SSC15 (Figure 3g).

Sequential detection of SCP1 and SCP3 carried out on 82 cells revealed three configurations (Table 1). In 12% of the spermatocytes (10 cells), a quadrivalent with fully synapsed chromosomes, i.e., presence of the SC lateral and central elements all along the SSC1 and SSC15 axis, was observed (Figure 3d). In 37% of the spermatocytes (30 cells), partial asynapsis was observed on the quadrivalents in the breakpoint regions (Figure 3e). Finally, an association between the quadrivalent and the XY body was observed in 51% of the spermatocytes (42 cells) (Figure 3f).

### 3.4. Gene Expression Analysis

**Figure 4:**
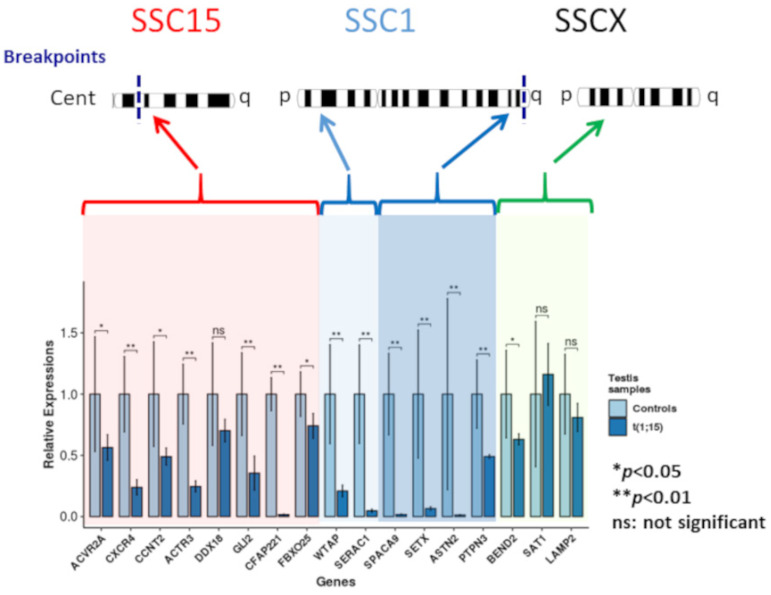
Gene expression analysis.

Downregulation compared to normal controls was observed for the genes located on SSC1 and SCC15, regardless of their localization on these chromosomes (Figure 4). Even if gene downregulation was expected in the breakpoint regions, it was not necessarily the case for genes located on the p arm of SSC1.

Downregulation of two of the three genes selected on the SSCX (statistically significant for *BEND2*) and upregulation for *STA1* (not statistically significant) were observed.

Relative expressions of the analyzed genes for the controls and the translocated boar (light and dark blue, respectively). Red highlighting: genes located on the centric region of chromosome 15; light blue highlighting: genes located on the p arm of chromosome 1; dark blue highlighting: genes of the q arm of chromosome 1; and green highlighting: chromosome X genes (* *p*-value < 0.05; ** *p*-value < 0.01).

## 4. Discussion

The meiotic behavior of chromosomal rearrangements has been investigated in a limited number of cases in pigs. Indeed, until now, meiotic analyses concerned only three Y-autosome [35,38,39] and one autosome–autosome translocations in this species [31]. Y-autosome translocations lead to an association of autosomes with the XY body, resulting in a total arrest of spermatogenesis.

Here, we analyzed the impact on the meiotic silencing process of an autosome–autosome translocation associated with azoospermia. To our knowledge, it is the first case of autosome–autosome translocation associated with azoospermia identified in pigs.

Such a phenotype seems very rare in this species as compared to humans. In pigs, heterosynapsis (pairing of nonhomologous chromosome fragments), avoiding apoptosis and subsequent meiotic arrest, seems to be relatively frequent during the early phases of pachytene [40]. This could explain the relatively low frequency of reciprocal translocations associated with defects in spermatogenesis observed in this species, as compared to humans [3].

Indeed, among more than 50,000 boars analyzed and more than 250 chromosomal rearrangements identified in our laboratory, of which 87% were reciprocal translocations, only two rearrangements seemed responsible for spermatogenesis disorders. These were the t(1;14) translocation associated to oligoasthenoteratospermia [31] and the t(1;15) translocation presented in this article. In both cases, the translocations were highly asymmetric (large difference in size of the exchanged chromosomal fragments). This characteristic led in both cases to chromosome pairing problems during the prophase of the first meiotic division.

Analysis of spermatocytes from the t(1;15) boar revealed a highly disturbed meiotic process. In fact, normal pairing of the normal and rearranged chromosomes 1 and 15 was observed in a limited number of cells (12% in the γH2AX experiment and 12% in SCP1 and SCP3 sequential staining).

Conversely, numerous meiotic pairing abnormalities were identified in 88% of the spermatocytes. Indeed, the presence of γH2AX or partial asynapsis without any association with the XY-body was detected on more than one third of the quadrivalents studied (34% in the γH2AX experiment and 37% in SCP1 and SCP3 sequential staining). An association of the quadrivalent with the XY body was observed in the majority of the spermatocytes with pairing troubles (54% in the γH2AX experiment and 51% in SCP1 and SCP3 sequential staining). Such phenomena have already been described in humans [18,26,28], mice [15] and pigs [31]. The frequency of this association increases along the pachytene substages and reaches a maximum at late pachytene [28]. It was proposed that this association could result from random chromatin movements of regions transcribed but not involved in the rearrangement [29]. It was also observed that the meiotic silencing of unpaired chromatin (MSUC) precedes and interferes with the MSCI and disturbs the transcriptional inactivation of the sex chromosomes as a trigger of apoptosis, resulting in spermatogenic arrest [15].

In order to confirm the gene silencing of unpaired chromatin hypothesized from the immunocytology experiments, gene expression analysis was carried out for genes located on SSC1 and SCC15, some of which were involved in the meiotic or spermatogenesis process. All these genes were downregulated compared to normal control boars. These data for gene expression concord with previous observations reported in mice [15]. Indeed, the autosome–autosome translocation studied by these latter authors was characterized by transcriptional downregulation of the genes inside the unsynapsed region of the rearranged mouse autosomes. They are also consistent with our previous study on an oligoasthenoteratospermic boar that carried a reciprocal translocation between chromosome 1 and 14 [31]. As reported in mice [15], the downregulation of genes in the unsynapsed autosomal chromatin occurs in pre-mid-pachytene spermatocytes, whereas a disturbance of transcriptional silencing of the X chromosome takes place later during the mid-late-pachytene stage. In our case, in contrast to the previously cited studies, we did not observe upregulation of the 3 SSCX genes analyzed, suggesting no or a limited impact of the translocation on MSCI.

However, this interpretation should be considered with caution, as the number of genes analyzed was very small. Gene expression analyses using microarray technology should be carried out to confirm these results.

However, we can suspect that the MSUC that occurs in early pachytene could have an impact on MSCI occurring in the latter pachytene stage.

Some of the genes studied are involved in the early step of spermatogenesis. *ACVR2A* contributes to the regulation of the entry in meiosis of embryonic germ cells [41]; *CCNT2* is involved in germ cell differentiation in the early or premeiotic steps of spermatogenesis [42]; *CXCR4* is involved in the maintenance of spermatogonial stem cells [43]; and *GLI2* sustains undifferentiated spermatogonial cells [44]. In order to detect a potential impact of the translocation on the spermatogonial cells, we analyzed the presence of these cells using immunohistology by detecting PGP9.5, a specific marker for undifferentiated spermatogonia in the porcine testis [45]. PGP9.5-positive spermatogonia lining the basal lamina of seminiferous tubules were observed in the same proportion as on a normal boar, suggesting no impact of the translocation at this stage.

Among the genes analyzed, some are involved in the late stage of spermatogenesis. Indeed, *CFAP221* is required for ciliary and flagellar function and primarily affects spermiogenesis [46]; *SPACA9* is involved in acrosome formation; and *ACTR3* (Arp3-actin-related protein 3) modulates actin dynamics within the seminiferous epithelium, in particular, at the apical ectoplasmic specialization and the blood–testis barrier [47].

In our case, meiotic arrest more especially concerns the spermatocyte stage, which suggests that there is no relationship between the expression alteration of the above-mentioned genes and the phenotype observed. Conversely, it can be postulated that the observed reductions in gene expression reflect more particularly the differences in testicular cell composition (absence of meiotic cells beyond the spermatocyte stage).

Among the genes analyzed, (Senataxin) *SETX* is particularly interesting according to the phenotype observed in the meiotic process of our animal: arrest at the spermatocyte stage and the presence of γH2AX foci on apparently synapsed autosomes. *SETX* (located at 272mb on SSC1) is very close to the SSC1 estimated breakpoint (274 mb). As mentioned in the introduction of this article, *SETX* is a DNA/RNA helicase involved in transcription regulation, RNA processing, and protection of the genome against oxidative damage. *SETX* is one effector involved the meiotic sex chromosome inactivation (MSCI), as well as in the interface of transcription and meiotic recombination. We observed a downregulation of the *SETX* in the translocated boar, as well as the presence of a γH2AX signal on apparently synapsed autosomes at the pachytene stage in 54.14% of the spermatocytes analyzed, just like in the spermatocytes of *Setx−/−* mice. The presence of γH2AX signals on chromosomal regions other than the ones involved in the rearrangement could also be correlated with a downregulation of genes on different chromosomes. This could explain the unexpected downregulation observed for the genes located on the p arm of the SSC1 chromosome. However, meiotic pairing analysis showed that a part of the spermatocytes (12% of the cells analyzed) present correct chromosome pairing without the presence of γH2AX signal. Therefore we are in a slightly different situation from the study performed on *Setx−/−* mice. Indeed, the *SETX* gene in our study is downregulated but not completely repressed. In these conditions it is not possible to conclude that the repression of *Setx* is the only cause of the observed azoospermia. An expression analysis from sorted cells could potentially answer this question but is not possible to carry out such an analysis from the frozen testicular samples we have.

## 5. Conclusions

In conclusion, reciprocal translocation t(1;15) was shown to strongly disturb the meiotic process (asynapsis, association between the quadrivalent and the XY body). A gene expression analysis confirmed that this rearrangement was responsible for meiotic silencing of unsynapsed chromatin (MSUC), but with no or a limited impact on MSCI. This result is particularly original. In contrast to the t(1;14) previously published [31], the meiotic process was totally arrested at the spermatocyte stage in our case, which suggests that MSUC could induce the downregulation of critical genes for spermatogenesis. The phenotype observed (total arrest of spermatogenesis and the presence of γH2AX foci on apparently synapsed autosomes) suggests that *SETX* downregulation could partially explain the azoospermia observed in this translocated boar.

## Figures and Tables

**Table 1:** Number of cells analyzed and percentage of the different pairing configurations observed in the immunocytology experiments.

	SCP3 + SCP1 + γH2AX Experiment			SCP3 + SCP1 Experiment		
	Normal Pairing	Asynapsis	Association with the XY Body	Normal Pairing	Asynapsis	Association with the XY Body
Number of cells	13	37	58	10	30	42
Total cells analyzed	108			82		
Percentage	12%	34%	54%	12%	37%	51%

## Data Availability

Data available on request due to restrictions eg privacy or ethicalThe data presented in this study are available upon request from the corresponding author. The data are not publicly available due to the ownership of the animal by a private company.

## Supplementary Materials

The following are available online at https://www.mdpi.com/article/10.3390/genes12081137/s1, Table S1: genes analyzed by real timePCR and they respective selected primers.
